# Magnetic Resonance Imaging-Based Morphometric Analyses of the Bicruciate Injury of the Knee: Is There a Clue in the Distal Femur?

**DOI:** 10.7759/cureus.56360

**Published:** 2024-03-18

**Authors:** Iffath Misbah, Praveen K Sharma, Afwaan Faizal, Paarthipan Natarajan

**Affiliations:** 1 Department of Radiology, Saveetha Medical College and Hospital, Saveetha Institute of Medical and Technical Sciences, Saveetha University, Chennai, IND

**Keywords:** knee dislocation, mri, knee dislocations, cruciate ligament injuries, femoral notch morphology

## Abstract

Background: The morphology of the distal femur's intercondylar notch has been implicated in the susceptibility to and severity of cruciate ligament injuries. While previous research has primarily focused on isolated anterior cruciate ligament (ACL) or posterior cruciate ligament (PCL) injuries, the relationship between notch morphology and combined cruciate injuries remains less understood.

Objective: This study aimed to explore the association between femoral notch morphology and the severity of combined cruciate ligament injuries in adult males.

Methods: In this retrospective cohort study, MRI scans from 118 adult male participants with and without knee dislocations (60 cases with Schenk classification Type II or higher knee dislocations and 58 controls) were analyzed. The study period ranged from 2015 to 2023. Femoral notch width, notch width index (NWI), and notch shape (U shape, A shape) were assessed using a Philips Multiva 1.5 Tesla system (Philips, Amsterdam, Netherlands). The statistical significance of differences in measurements between cases and controls was evaluated using independent sample t-tests performed with IBM SPSS Statistics, version 26 (IBM Corp., Armonk, NY).

Results: The case group exhibited a significantly smaller mean femoral notch width (15.88 mm ± 2.7 mm) and NWI (0.238 ± 0.58) compared to the control group (notch width 18.29 mm ± 3.4 mm, NWI 0.25 ± 0.31), with p-values of 0.004 for both measurements. The notch shape was predominantly A-shaped in the case group (n = 49) as opposed to U-shaped in the control group (n = 41).

Conclusions: The study identifies a significant association between reduced femoral notch dimensions and the severity of complex cruciate ligament injuries. These findings support the notion that specific femoral notch morphologies may predispose individuals to more severe ligamentous injuries.

## Introduction

Knee ligament injuries represent a significant concern within both athletic and general populations, affecting millions globally each year [1]. Among these, injuries to the cruciate ligaments, particularly the anterior cruciate ligament (ACL) and, to a lesser extent, the posterior cruciate ligament (PCL), are common and pose substantial challenges in terms of treatment and rehabilitation. The prevalence of such injuries poses not only a pressing health issue but also a considerable economic burden due to medical expenses and lost productivity [2,3].

The intricate biomechanics of the knee joint, along with its susceptibility to injury under excessive strain or during traumatic events, have led researchers to explore various anatomical and functional determinants of ligament injuries. One area of increasing interest is the morphology of the distal femur, particularly the geometry of the intercondylar notch where the cruciate ligaments are situated. Previous studies have suggested that variations in notch shape and size may predispose individuals to a higher risk of ligamentous tears, proposing a potential anatomical predisposition to such injuries. The distal femur notch morphology, including parameters such as notch width, notch width index, and notch shape, has been implicated in altering the biomechanical properties of the knee joint, influencing ligament tension, and potentially increasing the risk of injury under certain conditions [4,5].

Despite growing evidence of the correlation between distal femur notch morphology and cruciate ligament injuries, there remains a gap in the comprehensive understanding of this relationship. Many studies have focused on isolated aspects of notch morphology or have been limited by sample size, demographic diversity, or methodological inconsistencies [6-10]. Furthermore, the clinical implications of these findings, particularly concerning injury prevention, diagnosis, and treatment strategies, are yet to be fully elucidated.

This study aims to address these gaps by providing a detailed analysis of the distal femur notch morphology in a diverse cohort and assessing its impact on the prevalence of cruciate ligament injuries. Through advanced imaging techniques and a comprehensive methodological approach, we seek to clarify the extent to which distal femur morphology serves as a determinant of ligament injuries. By doing so, the research endeavors to contribute valuable insights that could inform preventive measures, enhance diagnostic accuracy, and guide therapeutic interventions, ultimately improving outcomes for individuals at risk of or suffering from knee ligament injuries.

## Materials and methods

This was a retrospective cohort study to investigate the association between distal femur notch morphology and the severity of cruciate ligament injuries in adult males. The study spanned from 2015 to 2023. The inclusion criteria for the cohort were as follows: adult males aged 18 to 60 years with MRI evidence of Type II or higher knee dislocation according to the Schenk classification system [10]. The exclusion criteria were as follows: all females, cases with fractures, and Type I knee dislocations to control for confounding factors that could affect the morphology of the distal femur notch.

Convenience sampling was employed to select participants from our database, which contains records of patients who underwent MRI scans for knee injuries during the study period. The total number of participants included in the study was 118, with 60 cases presenting with knee dislocations (Type II or higher) and 58 control subjects with normal MRI findings. Data collection was performed using Microsoft Excel (Microsoft Corp., Redmond, WA). Patient demographic data, as well as measurements of distal femur notch morphology, were extracted from medical records and imaging studies. The MRI scans were conducted using a Philips Multiva 1.5 Tesla system (Philips, Amsterdam, Netherlands).

**Figure 1:**
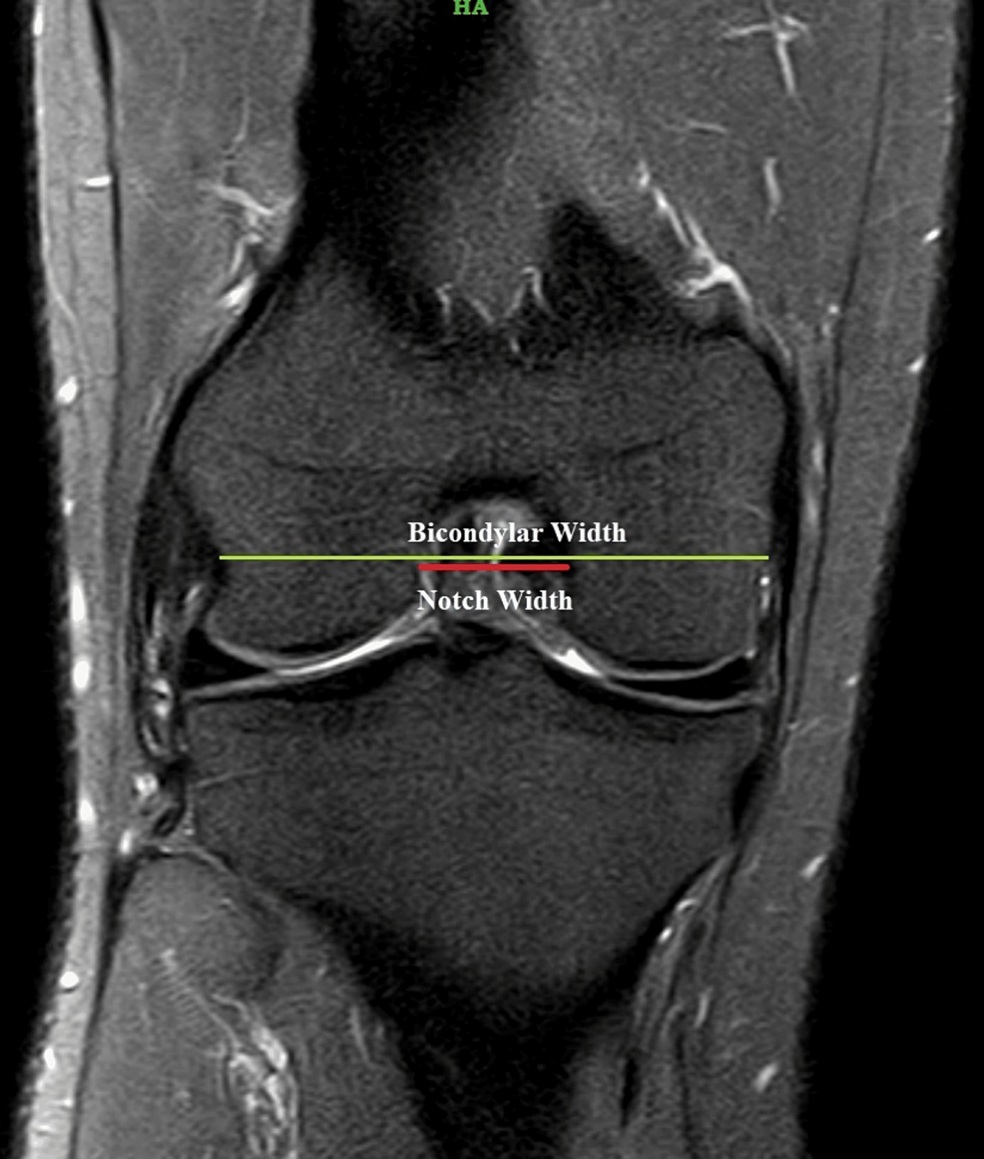
Coronal MRI image: proton density (PD) weighted fast spin echo (FSE) depicting the bicondylar width (green line) and notch width (red line)

**Figure 2:**
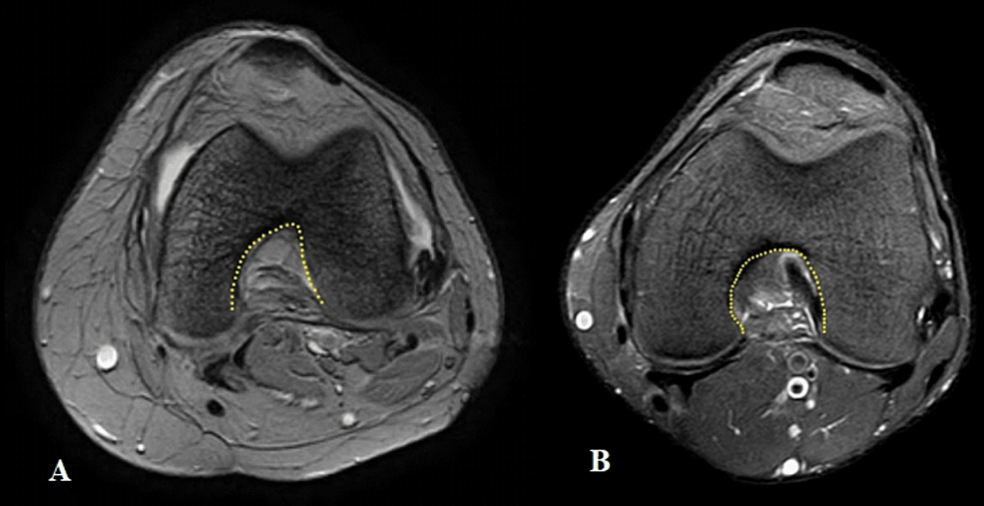
Axial MRI images: proton density (PD) weighted fast spin echo (FSE) sequence depicting the "A"-shaped notch (A) and the "U"-shaped notch (B), respectively.

A single observer recorded the radiological findings and measurements. Three primary dependent variables related to the femoral notch morphology were assessed in the proton density (PD) weighted spin echo sequence, which were: a) femoral notch width: the maximum width of the femoral notch was measured; b) notch width index (NWI): calculated by dividing the notch width at the level of the popliteal groove by the bicondylar width at the same level (Figure 1); c) notch shape: the shape (Figure 2) of the intercondylar notch was classified on the axial MRI sequence into one of two categories, namely U shape, where the notch width at the level of the popliteal groove (NWP) is equal to the notch width at the joint line (NWJ) within a 1 mm variance; and A shape, where the notch width is less than NWJ.

These morphological features were analyzed to identify any correlation with the severity of the cruciate ligament injuries determined by the Schenk classification.

Descriptive statistics were used to summarize the demographic characteristics of the participants. Independent sample t-test (Student's t-test) was performed using IBM SPSS Statistics, version 26 (IBM Corp., Armonk, NY) to test for significance between the notch measurements in cases and controls. A p-value of less than 0.05 was considered statistically significant.

## Results

Participant demographics

**Figure 3:**
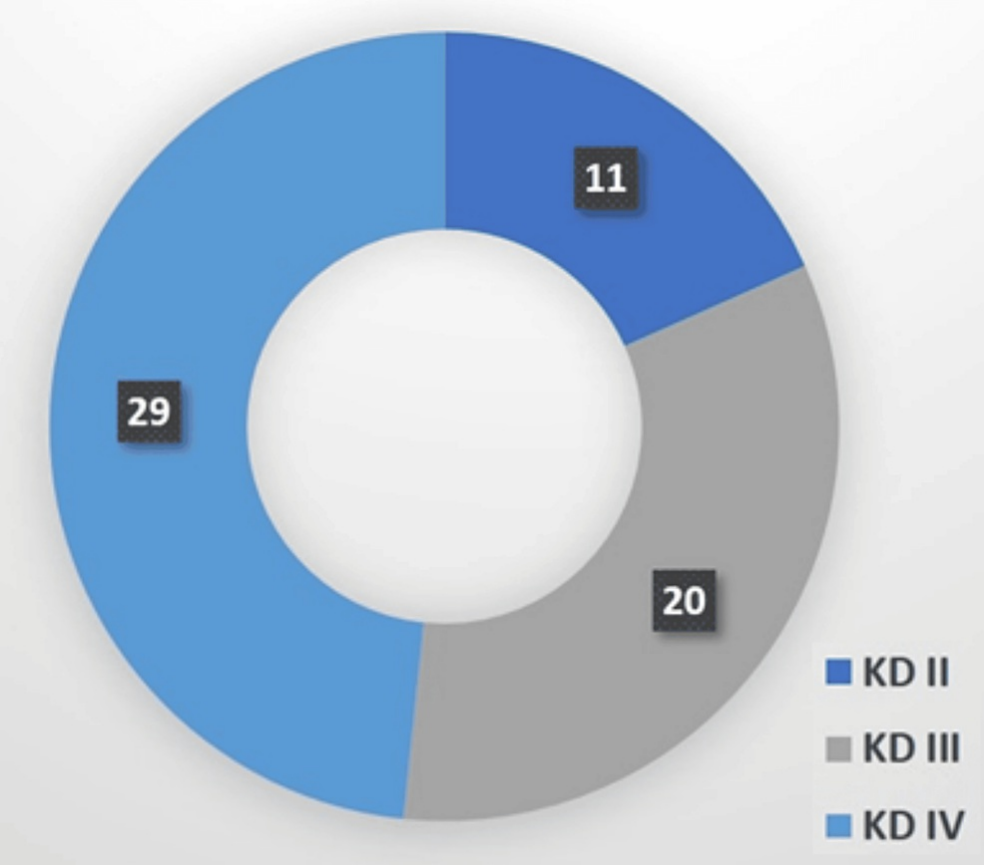
Distribution of knee dislocation (KD) types among the case group

The study comprised a case group of 60 individuals with knee dislocations and a control group of 58 individuals with normal MRI findings. The mean age was slightly higher in the case group (34 years, +/-4.2) compared to the control group (32 years, +/-3.4), indicating a young and active cohort across both groups (Table 1). The knee dislocations in the case group were classified using the Schenk system, revealing a distribution of 11 participants with Type II, 20 with Type III, and 29 with Type IV injuries (Figure 3). This classification helps in understanding the severity of the injuries among participants.

**Table 1:** Mean age of the study participants

Parameters	Control group	Case group
Total number of participants (n = 118)	58	60
Mean age	32 +/-3.4 years	34 (+/-4.2) years

Control group findings

This group included 58 individuals with normal MRI findings, serving as a baseline for comparison. The mean femoral notch width in this group was 18.29 mm with a standard deviation (SD) of 3.4 mm (Table 2). The NWI averaged 0.25, with a standard deviation (SD) of 0.31 (Table 2). These measurements are significant as they represent the normal range of femoral notch sizes. The shape of the intercondylar notch in most individuals (41 out of 58) in this group was U-shaped (Table 2).

**Table 2:** Results of femoral notch parameters A p-*v*alue of less than 0.05 was considered to be statistically significant.

Femoral notch parameters	Control group	Case group	p-value
Notch width (millimeter)	18.29 (+/- 3.4)	15.88 (+/- 2.7	0.004
Notch width index	0.25 (+/-0.31)	0.238 (+/- 0.58)	0.004
Notch shape	U (41/59)	A (49/60)	

Case group findings

In contrast to the control group, the case group (individuals with knee dislocations) showed a smaller mean femoral notch width of 15.88 mm (SD = 2.7 mm) (Table 2) and a lower mean NWI of 0.238 mm (SD = 0.58) (Table 2). Most individuals in this group (49 out of 60) had an A-shaped femoral notch (Table 2). These findings highlight significant differences in femoral notch morphology between individuals with knee dislocations and those with structurally normal knees.

Comparative analysis

Statistical analysis, specifically an independent sample t-test, revealed significant differences between the case and control groups in terms of femoral notch width and NWI, with p-values of 0.004 for both. A p-value of 0.004 suggests that the probability of these results occurring by chance is very low, hence they are statistically significant (Table 2).

The study revealed that individuals in the case group who had more severe knee dislocations often had a smaller femoral notch (horizontal stenosis). This observation indicates a potential relationship between the shape and size of the femoral notch (specifically, smaller notches) and the extent of damage to the cruciate ligaments. The prevalence of smaller notches in patients with more severe knee dislocations suggests that distal femoral morphometry plays a crucial role in the severity of these injuries.

## Discussion

The study revealed that a smaller femoral notch width and NWI are significantly associated with the severity of cruciate ligament injuries in adult males, with a notable prevalence of A-shaped notches in the case group. This finding is consistent with the hypothesis that specific morphological characteristics of the femoral notch may predispose individuals to cruciate ligament injuries, potentially through mechanisms such as mechanical impingement or altered biomechanical stresses on the ligaments.

The association between femoral notch morphology and ligament injuries, particularly the ACL, has been previously documented. Studies have suggested that a narrower notch may contribute to a higher risk of ACL injuries by creating a constricted space for ligament movement, leading to increased friction and wear [11-13]. Also, there have been radiological as well as anthropometric studies on the Indian population that have noted similar findings [8,14]. Fahim et al. noted a notch width of 19.9 mm ± 1.7 mm in the case group and 21.1 mm ± 1.9 mm in the control group; a NWI of 0.27 ± 0.01 in the case group; and 0.31 ± 0.01 in the control group [8]. Similarly, Park et al. noted that the mean value of notch width measured 17.37 mm in the ACL-injured patients, and 20.33 mm in the control population, with an NWI of 0.23 ± 0.01 in the case group and 0.24 ± 0.01 in the control group [7]. These findings are in concordance with the results observed in the present study. 

There have been isolated reports of PCL injuries due to decreased notch width. Apart from femoral notch indices, these studies have elucidated that the tibial slope also contributes to PCL injury severity [9,15,16]. Huang et al. noted a notch width of 20.1 mm ± 0.2 mm in their cohort [9]. The current findings extend these observations to more severe forms of knee injuries, including those involving multiple cruciate ligaments, thereby suggesting that the implications of notch morphology may be broader than previously recognized.

The notch's development is subjected to plastic deformation throughout an individual’s lifetime. A-shaped notches with narrow bases are found predominantly in childhood (<11 years), whereas U-shaped notches were noted in middle age (20-40 years) and omega-shaped notches in the elderly (>60 years) [17]. The predominance of A-shaped notches in individuals with more severe injuries further aligns with recent biomechanical models, which propose that certain notch shapes may alter the trajectory or tension of the cruciate ligaments during knee motion, thereby increasing the risk of injury under strain [4,6,9]. However, the relationship between notch shape and isolated PCL injuries remains less explored in the literature, highlighting an area for future investigation.

The insights from the present study have significant implications for clinical practice. Recognizing the role of femoral notch morphology in the risk of cruciate ligament injuries could enhance the screening and early identification of individuals at higher risk. For orthopedic surgeons, understanding these morphological risk factors may inform surgical planning, such as the consideration of notchplasty during ACL reconstruction in patients with narrower notches.

Moreover, the current study findings highlight the potential for developing personalized preventive strategies, including targeted strengthening exercises or bracing, to mitigate the risk of injuries in individuals with identified morphological predispositions.

The study's limitations include its retrospective design, reliance on a single radiologist's measurements, and the absence of consideration for injury severity beyond the Schenk classification. These factors may introduce biases and limit the generalizability of our findings. Future research should aim to include larger, more diverse populations and employ multiple observers to validate the measurements of femoral notch morphology.

Additionally, while the present study advances the understanding of the relationship between femoral notch morphology and cruciate ligament injuries, the mechanisms underlying this association remain to be fully elucidated. Prospective studies exploring the biomechanical impact of different notch shapes and sizes on ligament stress and strain during dynamic knee movements could provide valuable insights.

## Conclusions

In summary, the study highlights the significance of femoral notch morphology as a determinant of cruciate ligament injury severity, offering a novel perspective on the anatomical risk factors for these injuries. By integrating morphological assessment into the clinical evaluation of patients at risk for knee injuries, clinicians can better identify, manage, and possibly prevent the occurrence of these debilitating conditions.
